# Supplementary material for: Learning fast and fine-grained detection of amyloid neuropathologies from coarse-grained expert labels
Source: Commun Biol. 2023 Jun 24;6:668. doi: 10.1038/s42003-023-05031-6 (PMC10290693; doi:10.1038/s42003-023-05031-6)
Supplement: Supplementary file 1 — Supplementary Information [file 42003_2023_5031_MOESM1_ESM.pdf]

Supplementary Information

Learning fast and fine-grained detection of amyloid neuropathologies from coarse-grained expert labels

Daniel R. Wong[1,2,3,4,5], Shino D. Magaki[6], Harry V. Vinters[6,7], William H. Yong[8], Edwin S. Monuki[8], Christopher K. Williams[6], Alessandra C. Martini[8], Charles DeCarli[9], Chris Khacherian[8], John P. Graff[10], Brittany N. Dugger[10*], Michael J. Keiser[1,2,3,4,5*]

1. Institute for Neurodegenerative Diseases, University of California, San Francisco, San Francisco, CA, 94158, USA
2. Bakar Computational Health Sciences Institute, University of California, San Francisco, CA, 94158, USA
3. Department of Pharmaceutical Chemistry, University of California, San Francisco, San Francisco, CA, 94158, USA
4. Department of Bioengineering and Therapeutic Sciences, University of California, San Francisco, San Francisco, CA, 94158, USA
5. Kavli Institute for Fundamental Neuroscience, University of California, San Francisco, San Francisco, CA, 94158, USA
6. Department of Pathology and Laboratory Medicine, University of California, Los Angeles, Los Angeles, CA, 90095, USA
7. Department of Neurology, David Geffen School of Medicine at University of California, Los Angeles, Los Angeles, CA, 90095, USA
8. Department of Pathology & Laboratory Medicine, University of California, Irvine, CA 92697, USA
9. Department of Neurology, School of Medicine, University of California-Davis, Davis, CA 95817, USA
10. Department of Pathology and Laboratory Medicine, School of Medicine, University of California, Davis, Sacramento, CA 95817, USA

*Correspondence: bndugger@ucdavis.edu, keiser@keiserlab.org

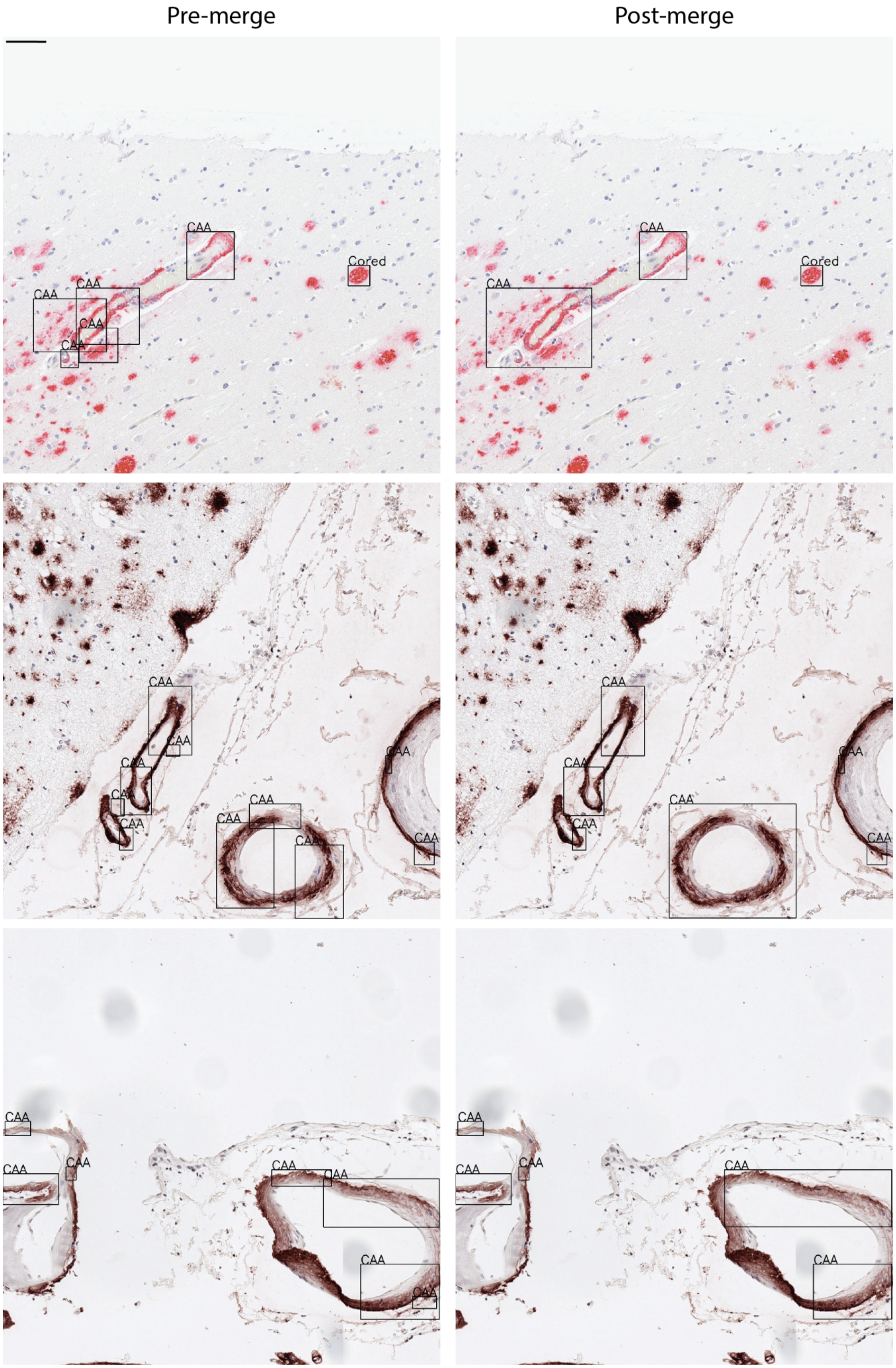
Pre-merge
Post-merge
CAA
Cored

Supplementary Figure 1: Comparing raw pre-merged labels with merged labels. Column left shows the raw bounding box labels. Box coordinates were derived from traditional water-shedding methods unassisted by human intelligence. For each identified class shown, at least two out of five expert annotators positively labeled the pathology. Column right shows the merged bounding box labels used to train model version one. Scale bar = 100µM.

a

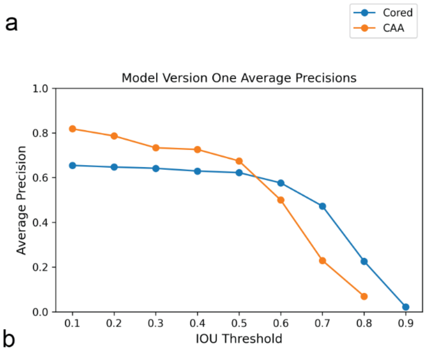

b

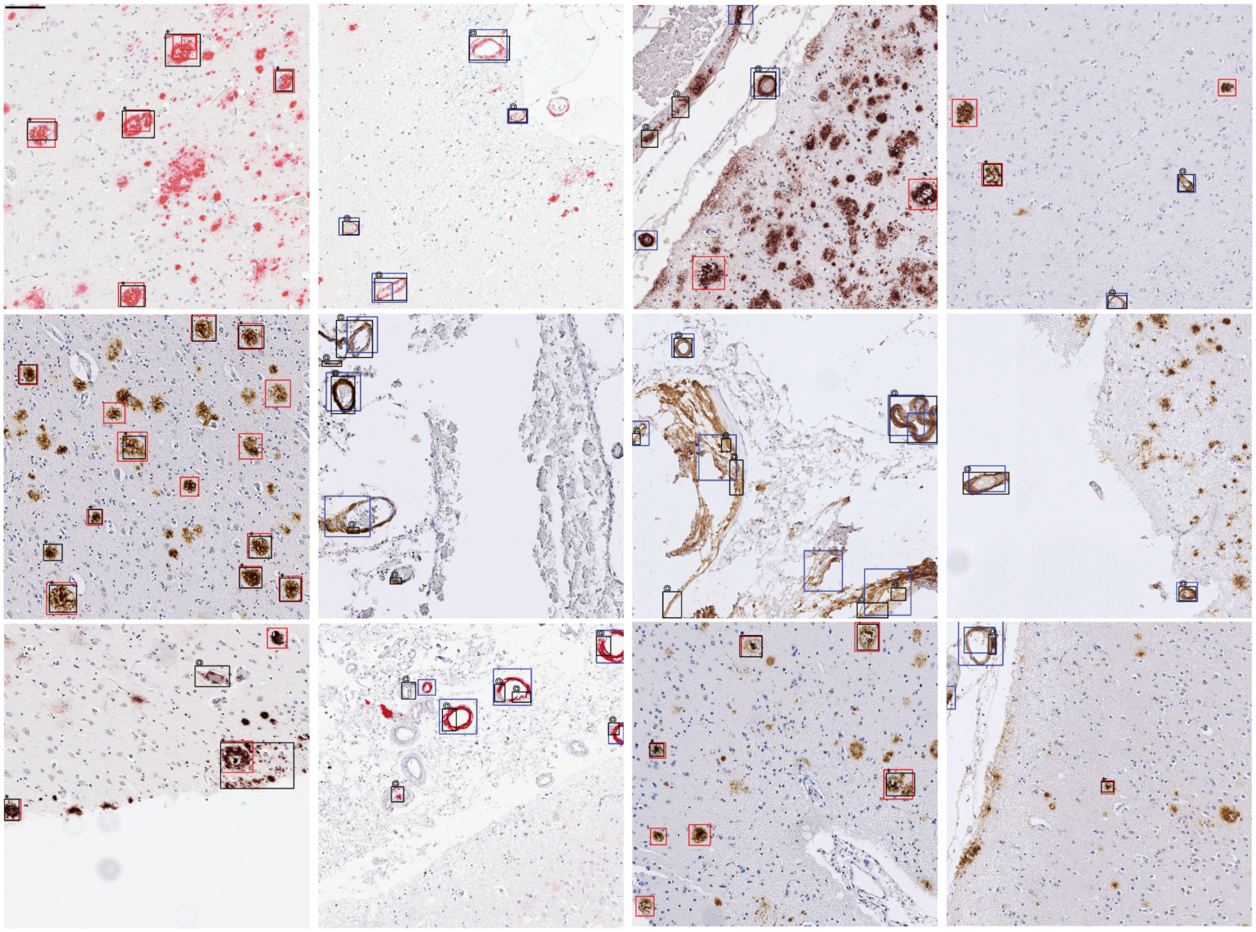

Supplementary Figure 2: Model version one performance and example image predictions. (a) Average precisions over the validation set for various IOU thresholds. The AP at IOU=0.90 is undefined for CAA. Positive annotation sample sizes for Cored = 1274, CAA = 256. (b) Example images are pulled from the validation set. Cored prediction: red, Cored label: black "*"; CAA prediction: blue, CAA label: black "@". It is important to note that the label data is sparse and does not contain every pathology (Methods). Scale bar = 100μM.

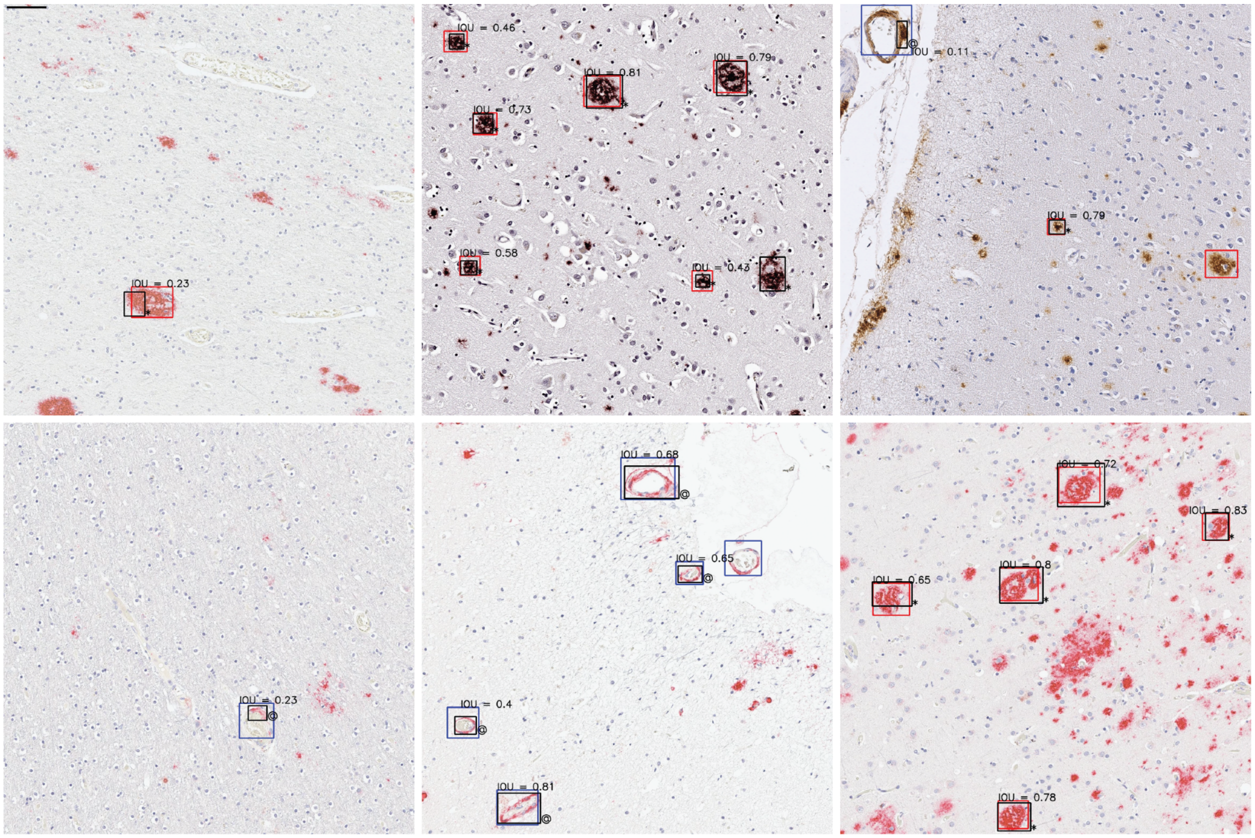

Supplementary Figure 3: Examples of different IOU values for overlaps. IOU values are shown for any overlaps between predicted bounding box (blue for CAA, red for Cored) and label bounding box (black). CAA labels are denoted by the "@" symbol, while Cored labels are denoted by the "*" symbol. Scale bar = 100μM.

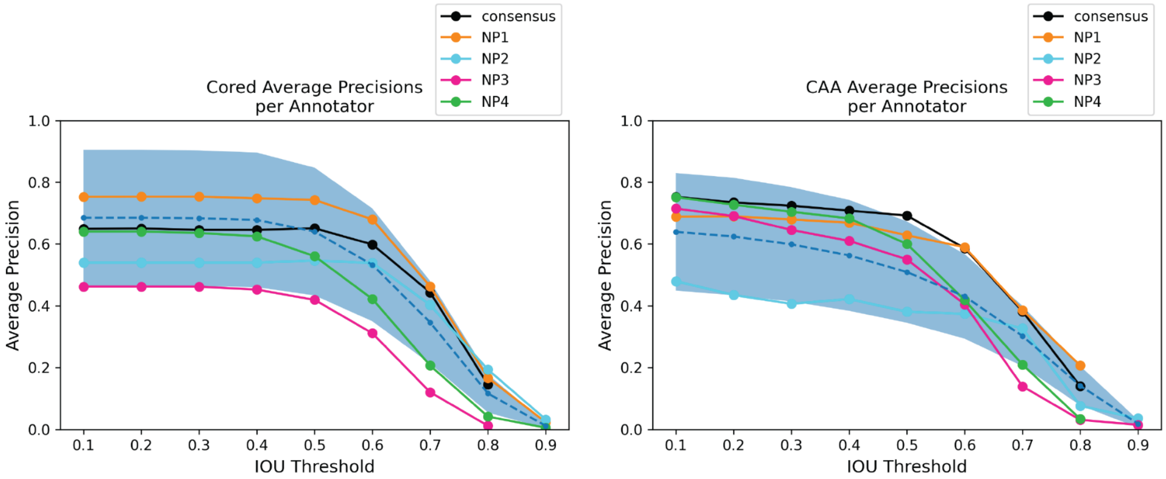

Supplementary Figure 4: Model version one also achieved human-expert level performance at identifying cored and CAA pathologies. Average model precision scores for identifying cored pathologies (left) and CAA pathologies (right). Y-axis: average precision, x-axis: IOU threshold that determines the minimal IOU required for a prediction to overlap with a label to be a true positive. Higher IOU thresholds are more stringent. The figure legend indicates which of the annotators is the ground truth benchmark for assessing the model. The black line indicates model AP against the consensus annotator benchmark. The blue dotted line is the average precision of comparing expert annotators to each other (Methods). The blue-shaded region is one standard deviation above and below the average-expert precision. Sample sizes (ground truth, Cored positive annotations, CAA positive annotations): (NP1, 370, 259), (NP2, 153, 153), (NP3, 121, 395), (NP4, 235, 324), (consensus with IOU = 0.50, 231, 289).

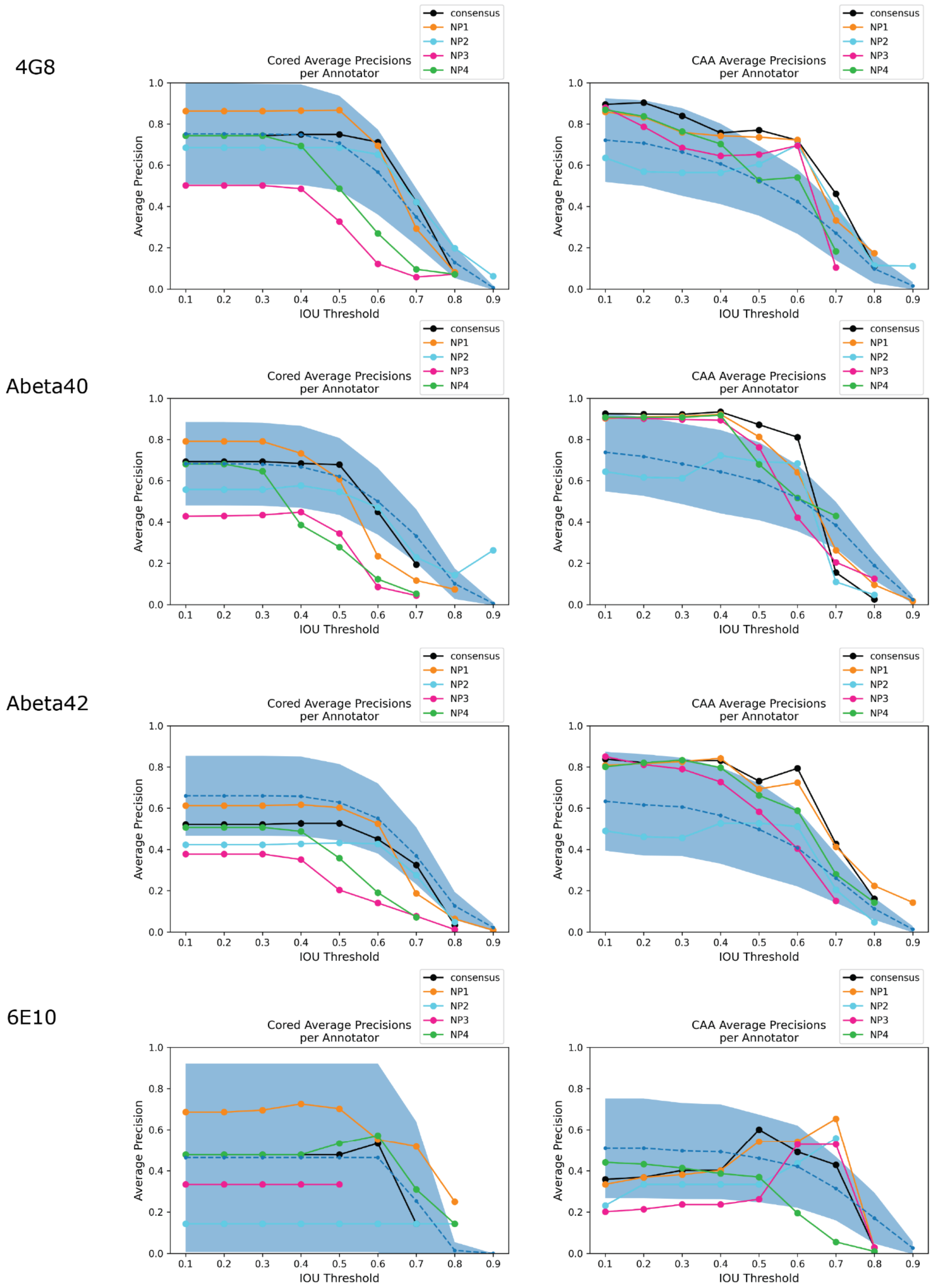

Supplementary Figure 5: Average precision by stain. We compute average precision on the

prospective validation set stratified by stain. At certain IOU thresholds, there are no true positive cases and correspondingly no precision scores. Sample sizes of consensus with IOU = 0.50 (stain, Cored positive annotations, CAA positive annotations): (4G8, 66, 48), (Abeta40, 72, 110), (Abeta42, 86, 88), (6E10, 7, 43).

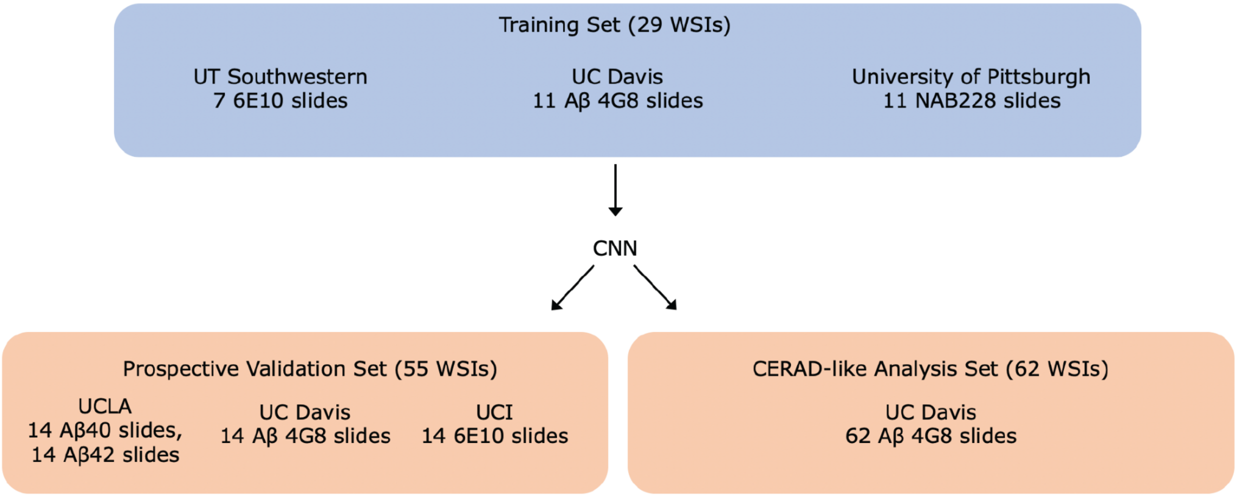

Supplementary Figure 6: Schematic of training and prospective validation datasets. We used a total of 29 WSIs from three institutions for training all models from Wong et al[1]. We used a separate new dataset of 55 WSIs from three institutions for prospective validation. To calculate comparison versus CERAD-like scores (Figure 4), we used a third dataset of 62 WSIs from Tang et al[2].

Architecture	x86_64
CPU op-mode(s)	32-bit, 64-bit
Byte Order	Little Endian
CPUs	64
Thread(s) per core	2
Core(s) per socket	16
Socket(s)	2
NUMA node(s)	2
Vendor ID	GenuineIntel
CPU family	6
Model	79
Model name	Intel(R) Xeon(R) CPU E5-2697A v4 @ 2.60GHz
Stepping	1
CPU MHz	1200.024
CPU max MHz	3600.0000
CPU min MHz	1200.0000
BogoMIPS	5200.02
Virtualization	VT-x
L1d cache	32K

Supplementary Table 1. CPU specifications.

Supplementary Note 1

Amyloid Beta Object Detection

Thank you for volunteering to be an annotator for this deep learning (DL) study that focuses on improving object detection, a critical initial input to developing models that will augment the ability of neuropathology experts!

Your annotations are invaluable, and necessary to validating our DL model which has shown a lot of promise. There are opportunities here to translate advances in the deep learning field to clinical research practice. The algorithm is not only fully interpretable, but also very fast and can even be run on a personal computer (no specialized hardware for deep learning needed!). We hope that this will make technological advances more accessible and equitable for novices and experts alike, and become highly impactful work that will be widely used.

We will be using the platform called "SuperAnnotate" to collect annotations. You should have received an invitation to the project. If not, please email dwong@keiserlab.org. There are 200 total images to annotate. Each image should take about 30 seconds to annotate, for a total time of 1 hour and 40 minutes for the whole project. All images should be annotated by **October 15, 2021.** Annotations can be completed either periodically, or all at once. Please see below for instructions on how to annotate. Thank you!

When you login, the landing page looks like this:

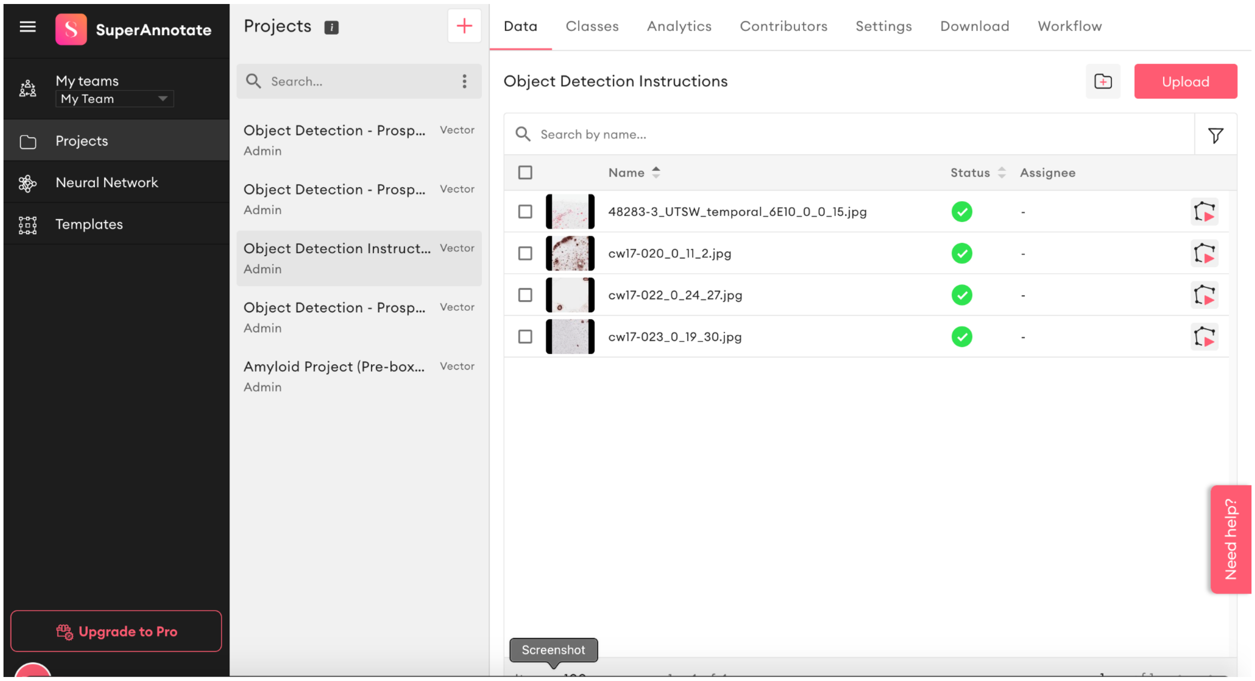

Click on the project entitled "Object Detection - Prospective Validation (YOUR NAME)". There should only be one project to select from. Select the first image to begin annotating. Once selected, the platform should look something like this:

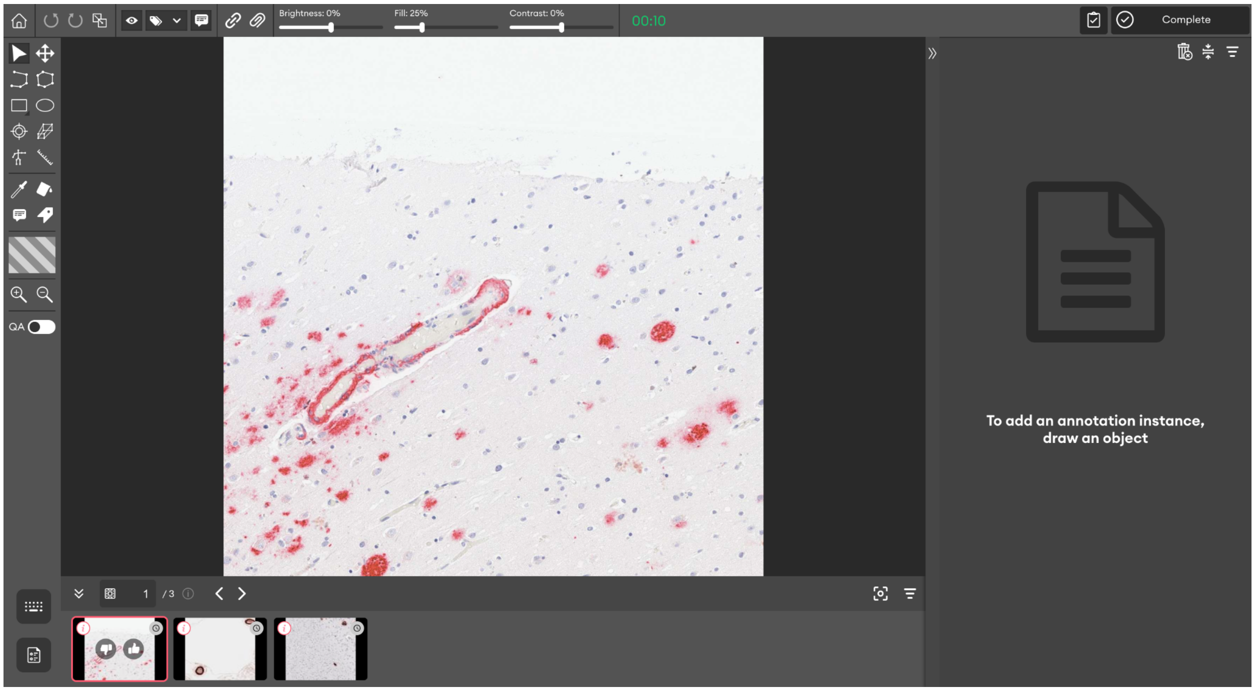

Instructions:

1) Identify **ALL Cored Amyloid-β plaques and CAA pathologies** within each image (ignore any other plaque types), and draw an appropriately labeled bounding box around each pathology. Try to capture the entire pathology within the box.

2) To draw a bounding box, you can click this square button indicated with a red arrow in the following image, or simply click the **"X"** key on your keyboard:

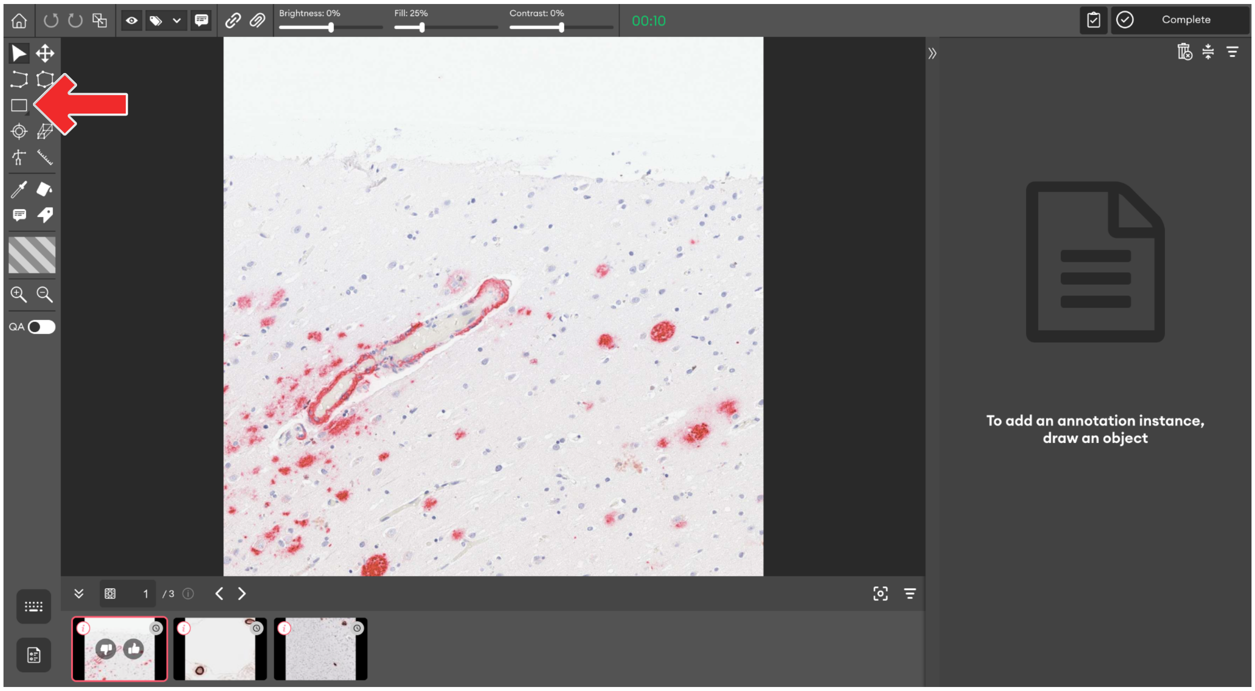

3) Now once the bounding box tool is selected, then simply click and drag to form a box that captures the entire pathology. It should look like this. After dragging to form a box, the box can be shifted by clicking the box and dragging it, or scaled by dragging the corners
   a) If a pathology is only partly within the image and the rest is out of frame, box as much of the pathology as you can.
   b) If there are two pathologies adjacent to each other, use separate boxes - one box for a single pathology.

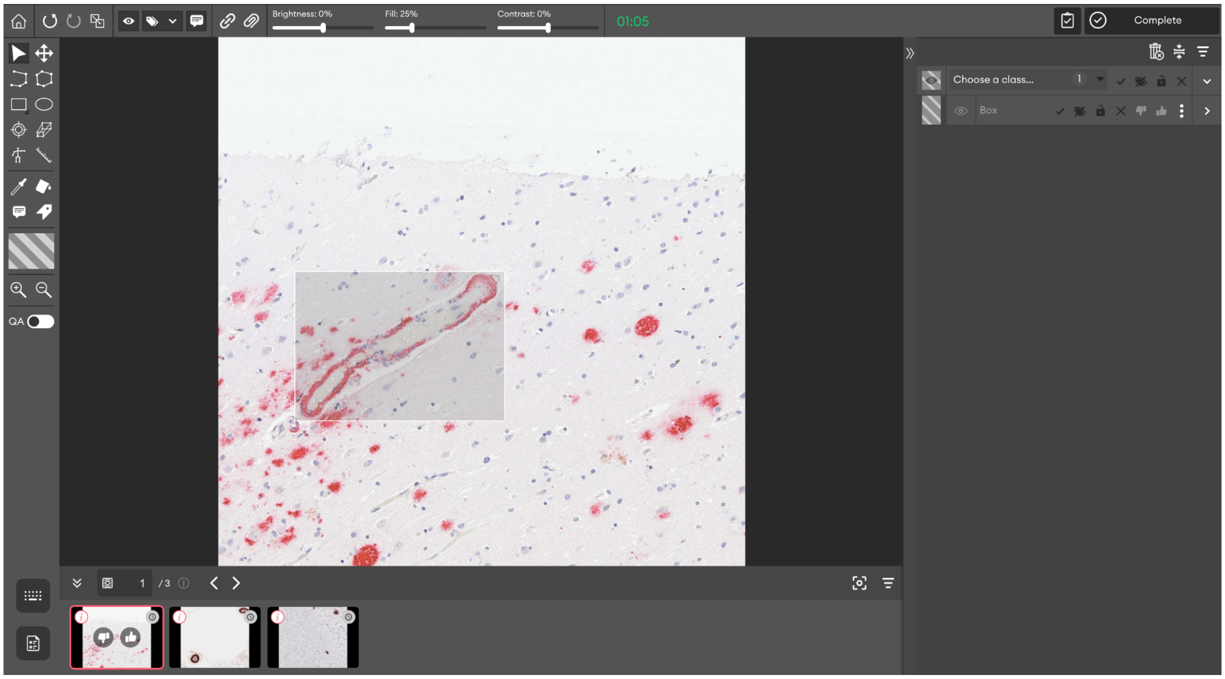

4) Once the box is drawn and captures as much of the pathology as you can, then right click on the box and select which class this pathology belongs to. Alternatively you can set the class by using the right panel and selecting "Choose a class":

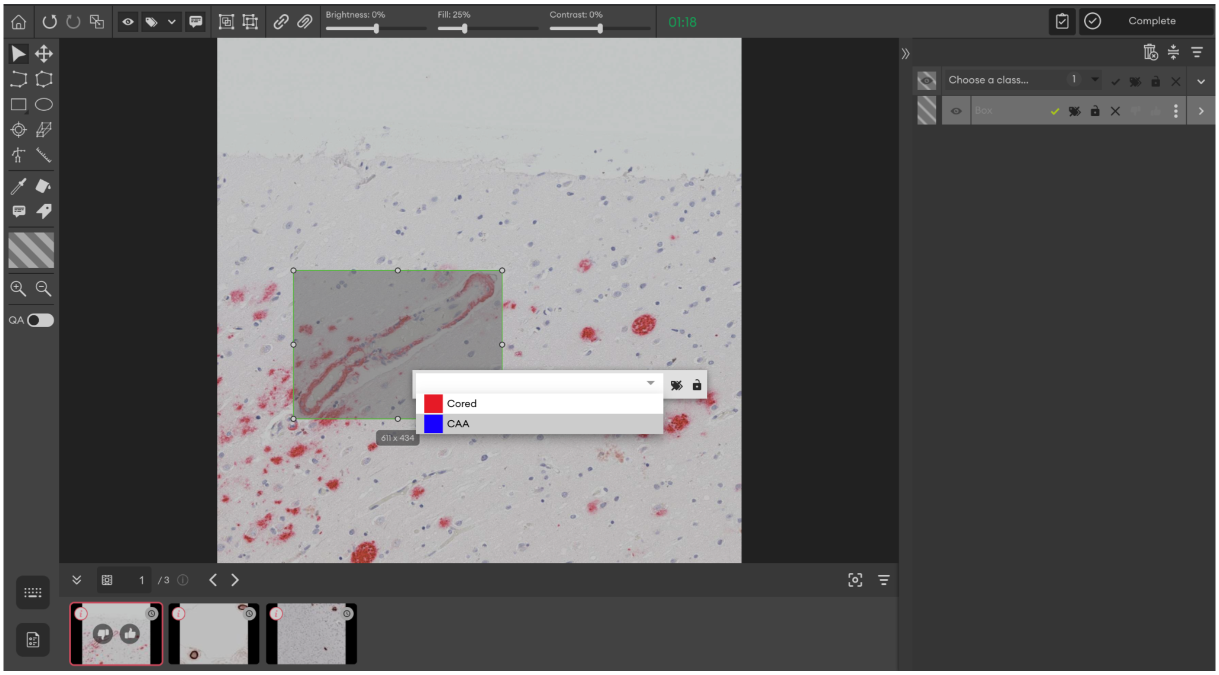

5) Once selected, the portal should look like this:

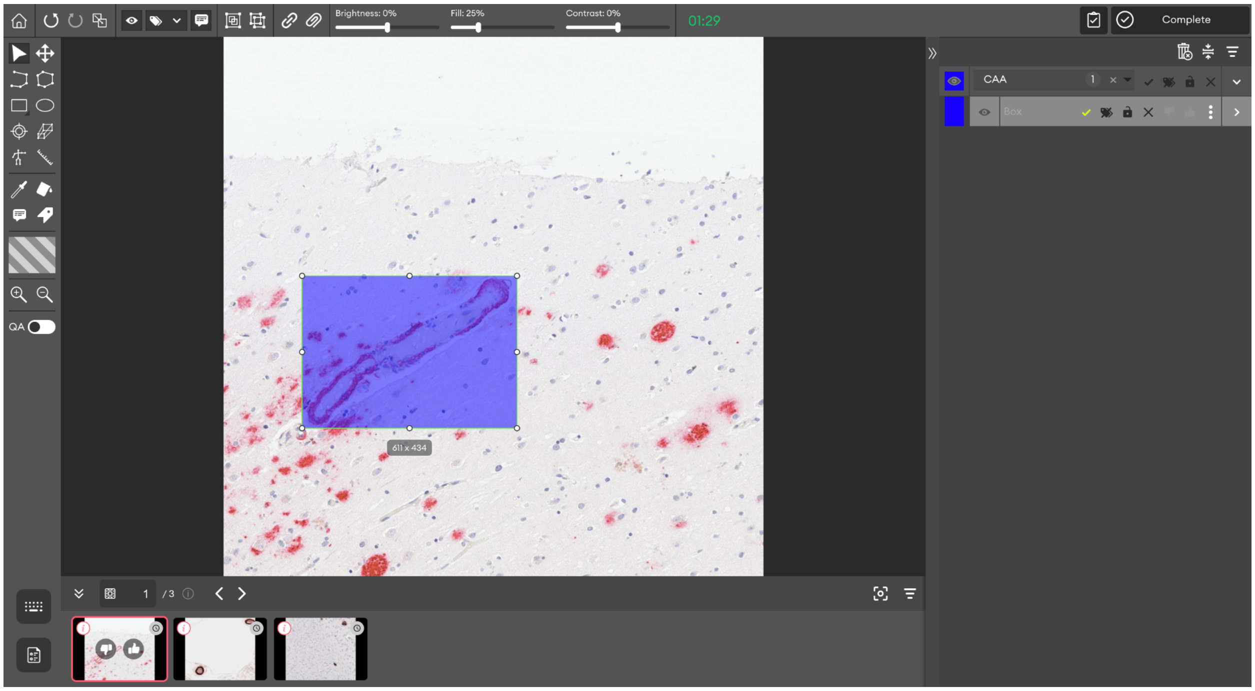

6) Repeat this process for each Cored or CAA pathology in the image.

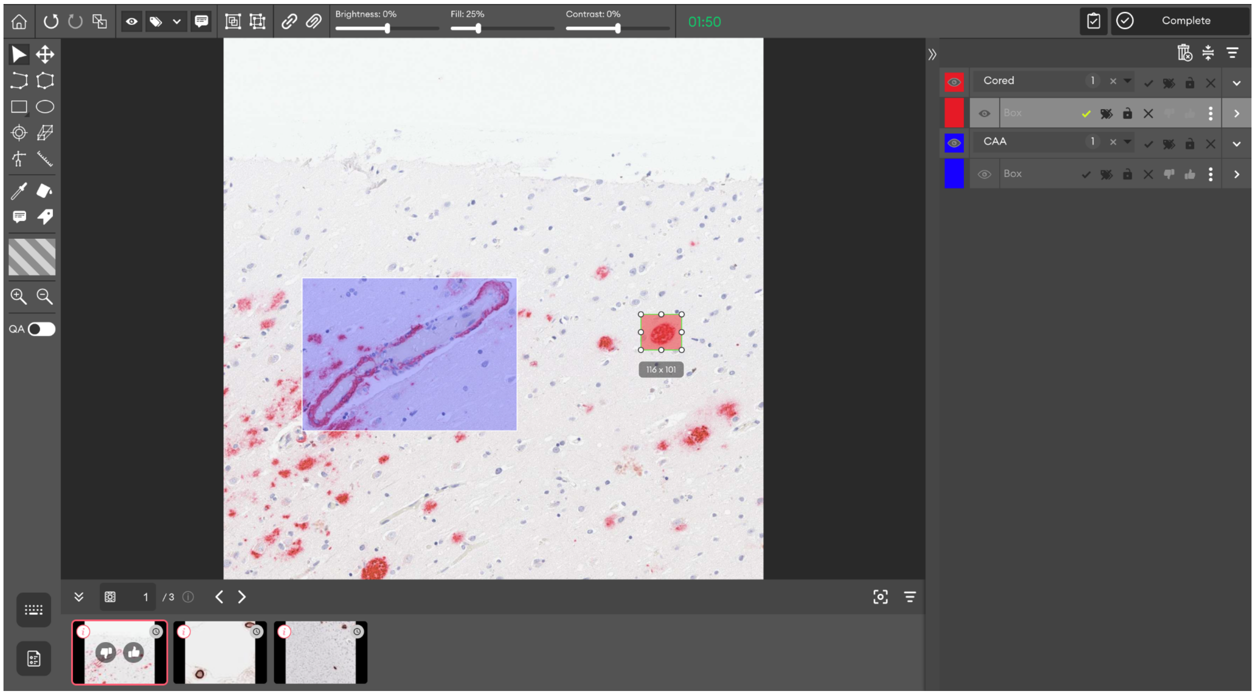

7) Once all pathologies have been captured by a labeled bounding box, move onto the next image.
8) Please make sure that all 200 images are annotated (or if no Cored or CAA pathology exists within an image, then leave the image blank and move onto the next)

More Examples

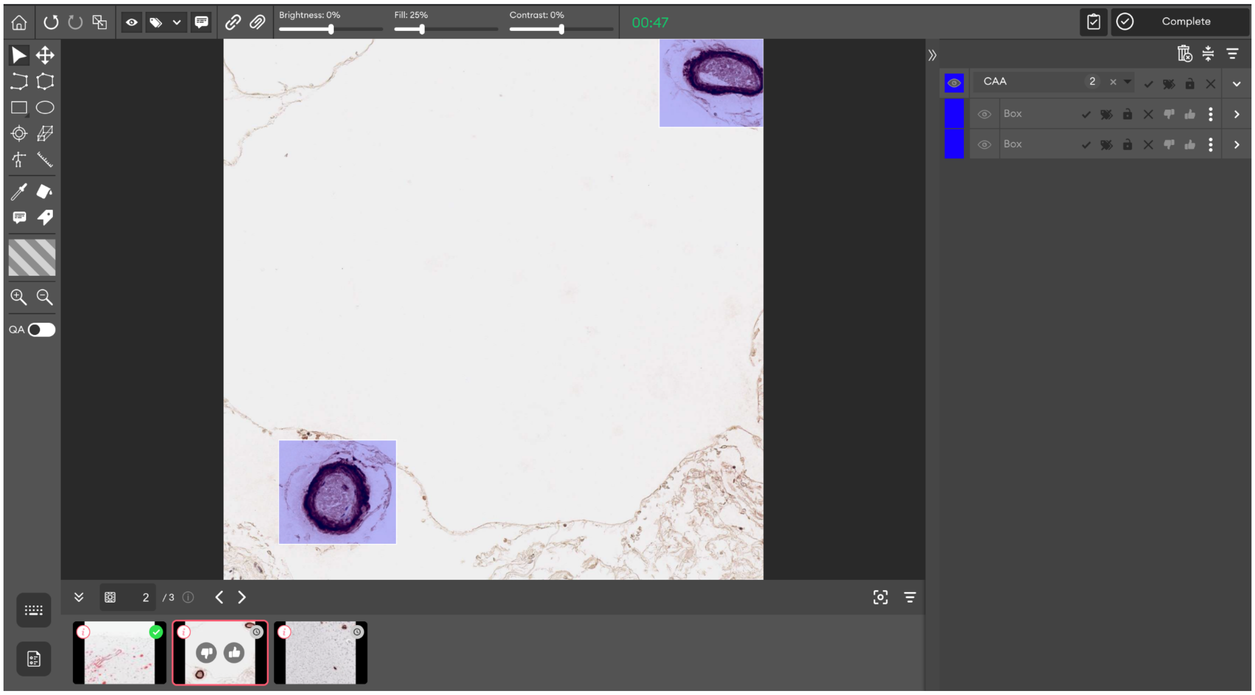

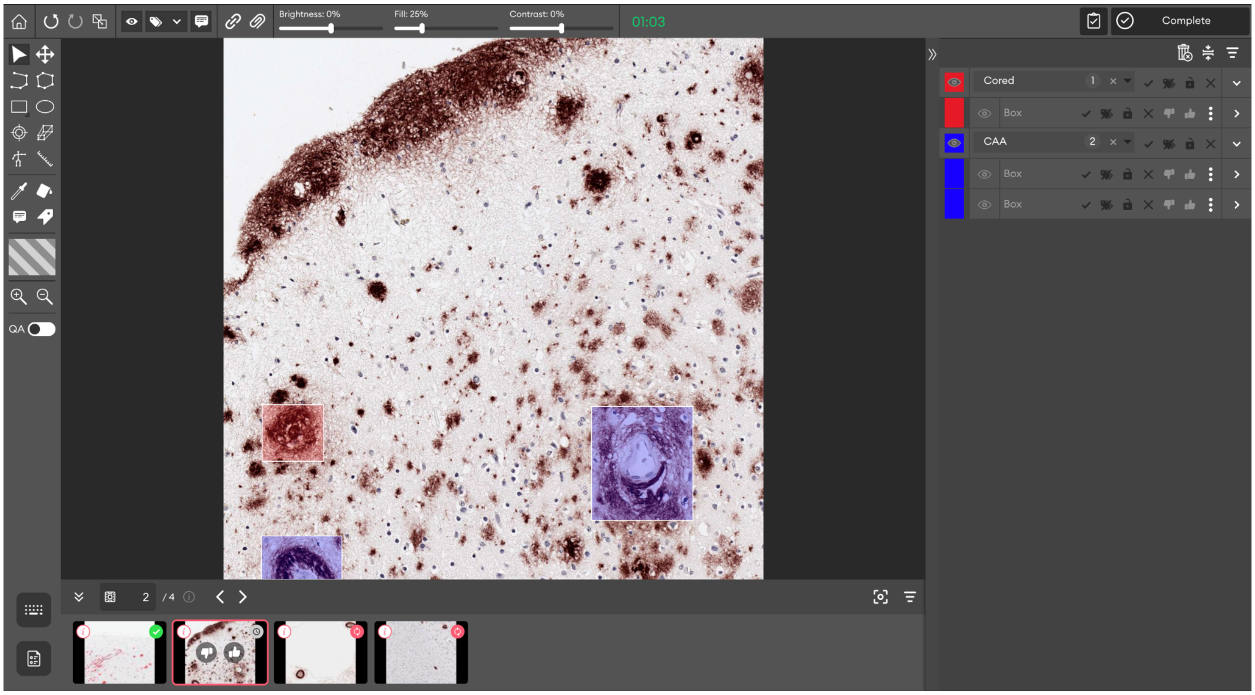

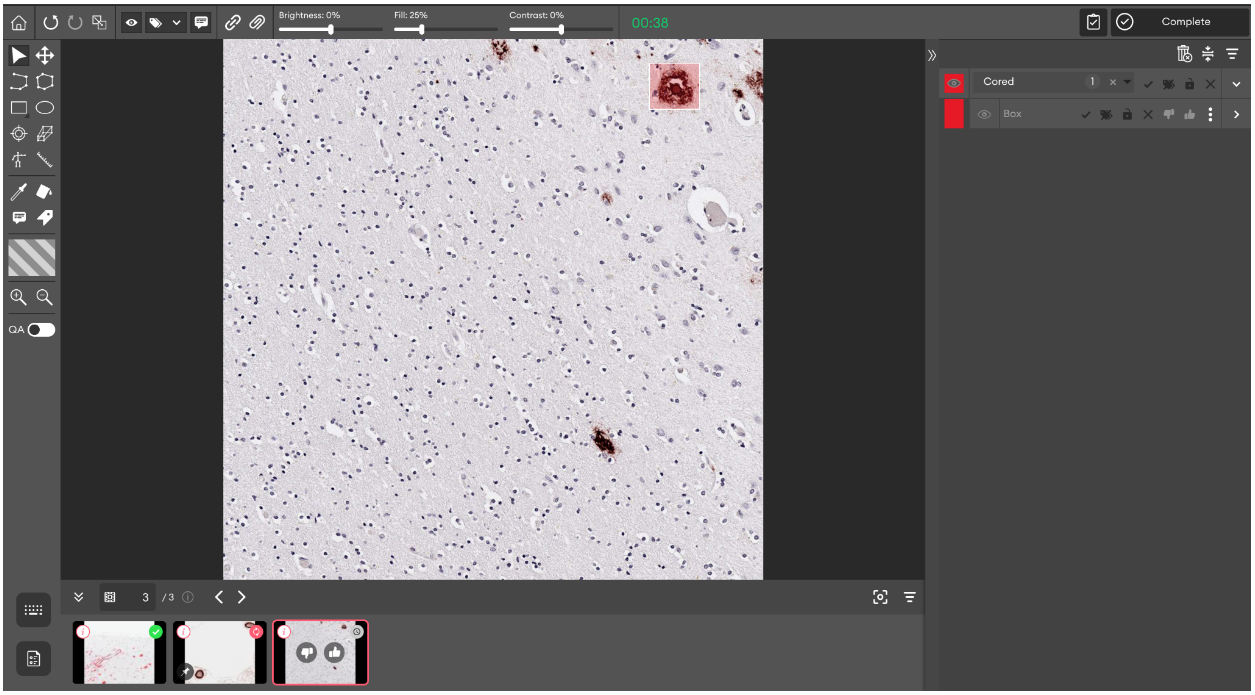

Other Useful Information:

- The image can be zoomed using the + and - magnifying glasses on the left tool panel
- The image can be panned using the “drag” button on the left tool panel
- To display all keyboard shortcuts, simply press “Ctrl” + “K” on your keyboard

Troubleshooting:

- If you run into any technical issues, try refreshing the page, or clicking the home button on the top left corner of the platform
- If any issues persist, please email dwong@keiserlab.org

Supplementary References

1. Wong, D. R. *et al.* Deep learning from multiple experts improves identification of amyloid neuropathologies. *Acta Neuropathol Commun* **10**, 66 (2022).
2. Tang, Z. *et al.* Interpretable classification of Alzheimer's disease pathologies with a convolutional neural network pipeline. *Nat. Commun.* **10**, 2173 (2019).
