# Supplementary material for: Learning fast and fine-grained detection of amyloid neuropathologies from coarse-grained expert labels
Source: Commun Biol. 2023 Jun 24;6:668. doi: 10.1038/s42003-023-05031-6 (PMC10290693; doi:10.1038/s42003-023-05031-6)
Supplement: Supplementary file 2 — Description of Additional Supplementary Files [file 42003_2023_5031_MOESM2_ESM.pdf]

Description of Additional Supplementary Files

File name: Supplementary Data 1

Description: Prospective dataset demographics and information
