# Supplementary material for: Learning fast and fine-grained detection of amyloid neuropathologies from coarse-grained expert labels
Source: Commun Biol. 2023 Jun 24;6:668. doi: 10.1038/s42003-023-05031-6 (PMC10290693; doi:10.1038/s42003-023-05031-6)
Supplement: Supplementary file 4 — Reporting Summary [file 42003_2023_5031_MOESM4_ESM.pdf]

nature portfolio

Corresponding author(s): Brittany N. Dugger and Michael J. Keiser

Last updated by author(s): May 23, 2023

Reporting Summary

Nature Portfolio wishes to improve the reproducibility of the work that we publish. This form provides structure for consistency and transparency in reporting. For further information on Nature Portfolio policies, see our Editorial Policies and the Editorial Policy Checklist.

Statistics

For all statistical analyses, confirm that the following items are present in the figure legend, table legend, main text, or Methods section.

n/a	Confirmed	
☐	☒	The exact sample size (*n*) for each experimental group/condition, given as a discrete number and unit of measurement
☒	☐	A statement on whether measurements were taken from distinct samples or whether the same sample was measured repeatedly
☐	☒	The statistical test(s) used AND whether they are one- or two-sided<br>*Only common tests should be described solely by name; describe more complex techniques in the Methods section.*
☒	☐	A description of all covariates tested
☒	☐	A description of any assumptions or corrections, such as tests of normality and adjustment for multiple comparisons
☐	☒	A full description of the statistical parameters including central tendency (e.g. means) or other basic estimates (e.g. regression coefficient) AND variation (e.g. standard deviation) or associated estimates of uncertainty (e.g. confidence intervals)
☐	☒	For null hypothesis testing, the test statistic (e.g. *F*, *t*, *r*) with confidence intervals, effect sizes, degrees of freedom and *P* value noted<br>*Give P values as exact values whenever suitable.*
☒	☐	For Bayesian analysis, information on the choice of priors and Markov chain Monte Carlo settings
☒	☐	For hierarchical and complex designs, identification of the appropriate level for tests and full reporting of outcomes
☒	☐	Estimates of effect sizes (e.g. Cohen's *d*, Pearson's *r*), indicating how they were calculated

Our web collection on statistics for biologists contains articles on many of the points above.

Software and code

Policy information about availability of computer code

Data collection	We used SuperAnnotate to collect validation data: https://www.superannotate.com/
Data analysis	We used Python for all of our data analysis. Code is custom and can be found at https://github.com/keiserlab/amyloid-yolo-paper.

For manuscripts utilizing custom algorithms or software that are central to the research but not yet described in published literature, software must be made available to editors and reviewers. We strongly encourage code deposition in a community repository (e.g. GitHub). See the Nature Portfolio guidelines for submitting code & software for further information.

Data

Policy information about availability of data

All manuscripts must include a data availability statement. This statement should provide the following information, where applicable:

- Accession codes, unique identifiers, or web links for publicly available datasets
- A description of any restrictions on data availability
- For clinical datasets or third party data, please ensure that the statement adheres to our policy

All de-identified image data are freely available at DOI: 10.17605/OSF.IO/FCPMW (https://doi.org/10.17605/OSF.IO/FCPMW).

Research involving human participants, their data, or biological material

Policy information about studies with human participants or human data. See also policy information about sex, gender (identity/presentation), and sexual orientation and race, ethnicity and racism.

Reporting on sex and gender	This study utilized whole slide images (WSIs) generated from select brain samples of human post-mortem tissues from a previously published report: Wong, D. R. et al. Deep learning from multiple experts improves identification of amyloid neuropathologies. Acta Neuropathologica Communications 10, (2022) and demographics are within that paper's Supplementary Figure S1. We additionally collected a new WSI dataset for Prospective Testing, whose demographics are listed in Supplementary File 1.<br><br>The study did not contain human subjects/participants, as only living subjects are defined as Human Subjects under federal law (45 CFR 46, Protection of Human Subjects). During life, all participants or legal representative signed informed consent as part of each Institutions' program. All human subject involvement was overseen and approved by the institutional review boards at each respective institution. All data will follow current laws/regulations and IRB guidelines (such as sharing de-identified data that does not contain information used to establish the identify of individual deceased subjects). De-identified data does not contain personal health information (PHI) like names, social security numbers, addresses, or phone numbers. Data is shared with a randomly generated pseudo-ID.<br><br>In summary, 55.8% of the first dataset was female and 44.2% male. 36.7% of the prospective dataset was female and 63.3% was male. However we do not calculate or report results specific to sex or gender.
Reporting on race, ethnicity, or other socially relevant groupings	See details of study dataset reported above, in "Reporting on sex and gender." We do not calculate or report results specific to race, ethnicity, or other socially relevant groupings.
Population characteristics	This study utilized whole slide images generated from select brain samples of human post-mortem tissues from a previously published report: Wong, D. R. et al. Deep learning from multiple experts improves identification of amyloid neuropathologies. Acta Neuropathologica Communications 10, (2022) and demographics are within that paper's Supplementary Figure S1. This study is considered a highly selected cohort and not a population. In summary 83.7% of the cohort were denoted as White, 2.3% Asian, 4.7% African American, and 7% of Hispanic descent.
Recruitment	This study utilized previously collected post-mortem samples that were obtained through Alzheimer's Disease Research Centers at the University of California Davis, University of Texas Southwestern, and University of Pittsburgh.
Ethics oversight	This study utilized already collected samples obtained through Alzheimer's Disease Research Centers (ADRC) at the University of California Davis, University of Texas Southwestern, and University of Pittsburgh. Each ADRC is governed by institutional IRBs. This study did not contain human subjects/participants, as only living subjects are defined as Human Subjects under federal law (45 CFR 46, Protection of Human Subjects). During life, all participants or legal representative signed informed consent as part of each Institutions' program. All human subject involvement was overseen and approved by the institutional review boards at each respective institution. All data will follow current laws/regulations and IRB guidelines (such as sharing de-identified data that does not contain information used to establish the identify of individual deceased subjects). De-identified data does not contain personal health information (PHI) like names, social security numbers, addresses, or phone numbers.

Note that full information on the approval of the study protocol must also be provided in the manuscript.

Field-specific reporting

Please select the one below that is the best fit for your research. If you are not sure, read the appropriate sections before making your selection.

☒ Life sciences ☐ Behavioural & social sciences ☐ Ecological, evolutionary & environmental sciences

For a reference copy of the document with all sections, see nature.com/documents/nr-reporting-summary-flat.pdf

Life sciences study design

All studies must disclose on these points even when the disclosure is negative.

Sample size	Samples were whole slide images. We used all of the images for training or validation and based the sample size off of our previous studies.
Data exclusions	No data were excluded.
Replication	All analyses are open source, and can be reproduced as specified in https://github.com/keiserlab/amyloid-yolo-paper.
Randomization	Randomization was applied during prospective validation. We randomly selected two WSIs that were different from the original 48. We randomly picked two fields for each of these eight WSIs. We chose this random subset in order to have a dataset small enough for humans to annotate manually.
Blinding	Annotators were blinded to experimental procedures and designs when labeling image data.

Reporting for specific materials, systems and methods

We require information from authors about some types of materials, experimental systems and methods used in many studies. Here, indicate whether each material, system or method listed is relevant to your study. If you are not sure if a list item applies to your research, read the appropriate section before selecting a response.

Materials & experimental systems

n/a	Involved in the study
☐	☒ Antibodies
☒	☐ Eukaryotic cell lines
☒	☐ Palaeontology and archaeology
☒	☐ Animals and other organisms
☒	☐ Clinical data
☒	☐ Dual use research of concern
☒	☐ Plants

Methods

n/a	Involved in the study
☒	☐ ChIP-seq
☒	☐ Flow cytometry
☒	☐ MRI-based neuroimaging

Antibodies

Antibodies used	APP/β-Amyloid (NAB228) Mouse mAb, Cell Signaling, #2450;<br>Anti-beta-Amyloid Purified (SIGNET) Monoclonal Antibody, Unconjugated, Clone 6E10, BioLegend, Cat# SIG-39320, RRID:AB_662798;<br>Anti-β-Amyloid, 17-24 Antibody, BioLegend, # SIG-39200
Validation	All antibodies were validated by the vendor. Documentation and relevant links here:<br>https://www.cellsignal.com/about-us/our-approach-process/cst-antibody-performance-guarantee<br>https://www.biolegend.com/en-us/products/purified-anti-beta-amyloid-1-16-antibody-11228<br>https://www.biolegend.com/en-us/products/anti-beta-amyloid-17-24-antibody-10999
